# The efficacy of esmolol, remifentanil and nitroglycerin in controlled hypotension for functional endoscopic sinus surgery

**DOI:** 10.1016/j.bjorl.2019.08.008

**Published:** 2019-10-03

**Authors:** Aslı Alkan, Mehtap Honca, Ali Alkan, Handan Güleç, Eyüp Horasanlı

**Affiliations:** a Muğla Sıtkı Koçman University Education and Research Hospital, Department of Anesthesiology and Reanimation, Muğla, Turkey; b Yozgat Bozok University School of Medicine, Department of Anesthesiology and Reanimation, Yozgat, Turkey; c Muğla Sıtkı Koçman University School of Medicine, Department of Internal Medicine, Division of Medical Oncology, Muğla, Turkey; d Ankara Yıldırım Beyazıt University, School of Medicine, Department of Anesthesiology and Reanimation, Ankara, Turkey

**Keywords:** Remifentanil, Esmolol, Nitroglycerin, Functional endoscopic sinus surgery, Controlled hypotension

## Abstract

**Introduction:**

Controlled hypotension is a reversible procedure in which the patient’s baseline mean arterial blood pressure is reduced by 30% and sustained at 60–70 mmHg during the procedure. It decreases blood loss and provides clear surgical field during the procedures.

**Objectives:**

The purpose of this study was to compare the efficacy of controlled hypotension agents esmolol, remifentanil, and nitroglycerin in functional endoscopic sinus surgery, in terms of hemodynamic changes and impact on the surgical efficiency.

**Methods:**

The research was carried out as a cohort study. Patients who underwent functional endoscopic sinus surgery were randomized into 3 groups. Controlled hypotension was achieved with remifentanil (Group R), esmolol (Group E) and nitroglycerin (Group N). The efficacy of the drugs was tested by comparing the length of time with the targeted mean arterial pressure, the amount of anesthetics used, surgical field bleeding score and surgeon’s satisfaction.

**Results:**

Between May to December 2015, 60 patients were included and randomized equally into 3 different study groups. The median of the length of time with the targeted mean arterial pressure was shorter in the Group R when compared with Group E (*p* = 0.01) and Group N (*p* = 0.14). The amount of volatile anesthetics used was 25.0 mL (15–51), 43.0 mL (21–105) and 40.0 mL (26–97) in Groups R, E and N, respectively (*p* < 0.001). While there was more bleeding with nitroglycerin, surgical field bleeding scores were lower in Group R when compared with esmolol (*p* = 0.001) and nitroglycerin (*p* < 0.001). The analysis of surgeon’s satisfaction scores concluded that surgeons were more satisfied with the group R (100%), when compared with group E (60%) and group N (30%) (*p* < 0.001).

**Conclusion:**

Less volatile agent, short time to achieve controlled hypotension, stable blood pressure, lower surgical field bleeding scores and larger length of time with the targeted mean arterial pressure were found as the advantages of Remifentanil. Less costly, efficiency of achieving the targeted median arterial pressure and less postoperative complications were the advantages of nitroglycerin. In functional endoscopic sinus surgery procedures, appropriate controlled hypotensive agents should be selected according to the patients’ characteristics and advantages/disadvantages of the drugs.

## Introduction

Functional Endoscopic Sinus Surgery (FESS) is an important way of approach, mostly used in pathologies obstructing the sinuses. In addition to its efficacy, the procedure provides drainage of sinuses without damaging the normal physiology and anatomy of the nasal cavity.^1^ The FESS is generally used for the treatment of nasal polyposis, recurrent acute rhinosinusitis, leakage of cerebrospinal fluid, fungal infections, foreign objects in the nasal cavity, mucocele, periorbital abscess, epistaxis and tumors.^2^ The main complication of the procedure is bleeding. The bleeding can decrease the quality of FESS and increase the risk of complications. Good control of bleeding provides better surgical success, less surgical trauma and short surgical time.^3^ For bleeding control, hypotensive measures are generally practiced, such as appropriate patient positioning, positive pressure ventilation, and hypotensive agents.

Controlled Hypotension (CH) at a moderate level is defined as a reversible and controlled reduction of Mean Arterial Pressure (MAP) to 60‒70 mmHg or a 30% reduction of baseline MAP.^4^ Numerous agents are used for CH. Volatile anesthetics, opioids, sodium nitroprusside, nitroglycerin, hydralazine, trimethaphan, adenosine, α-2 blockers and β-blockers are the most commonly used drugs for CH.^5^ There are both pros and cons for the use of these agents.

The purpose of this study is to compare the CH agents Esmolol, Remifentanil, and Nitroglycerin for FESS, in terms of hemodynamic changes and impacts on surgical efficacy.

## Methods

The study was conducted in Keciören Education and Research Hospital, Department of Anesthesiology and Reanimation. Institutional Ethics Committee approved the study protocol (nº B.10.4.İSM.4.06.68.49) and the study was in accordance with the ethical standards laid down in the 1964 Declaration of Helsinki. All participants gave their informed consent prior to their inclusion in the study.

Between 2015 May and 2015 December, ASA I–II patients, between the ages of 18‒50 and who were scheduled for FESS were included in the study. The patients with ASA score of III and more, medications including β-blockers, opioids and ones having impact on the cardiovascular system, chronic hypertension, coronary artery disease, arrhythmias, chronic obstructive pulmonary disease, chronic renal or hepatic failure, history of cerebrovascular disease, diabetes, severe anemia (less than 7 gr/dL), coagulopathy, history of sinus surgery, history of allergy to one of the study drugs and who were pregnant or lactating were excluded from the study. Sealed envelopes were used to assign the patients to treatment groups; Group R (remifentanil), Group E (esmolol) and Group N (nitroglycerin). The patients were randomized after signed a written informed consent just before the procedure. Two anesthesiologists followed the patients: one for randomization and preparation of the drug, one for monitoring and documentation of the results. Thus the observer was blinded.

The patients’ gender, age, weight, ASA score and indications of the FESS were recorded. Patients were monitored by ECG, pulse oximetry, invasive/noninvasive blood pressure and Bispectral Index (BIS). Thus Heart Rate (HR), systolic arterial pressure, diastolic arterial pressure, Mean Arterial Pressure (MAP), Oxygen Saturation (SpO_2_) and Bispectral Index (BIS) (Quatro TM, Aspect Medical System, Newton MA, USA) were recorded. After recording preoperative measurements, induction of anesthesia was performed with 1 mg/kg lidocaine (2%), propofol 2–3 mg/kg and fentanyl 1 μg/kg IV. Patients were intubated with 0.6 mg/kg rocuronium and maintenance of anesthesia was sustained by with sevoflurane 2–4%, nitrous oxide 50% and oxygen 50% to keep the BIS in the 40‒60 range. In addition, mechanical ventilation was continued to provide an end-tidal carbon dioxide level of 32–36  mmHg. All the patients were in 45 degrees supine position. Before starting the procedure CH was performed in study groups. In Group R, remifentanil was used with a loading dose of 1 mcg/kg in 60 sec. The maintenance of anesthesia was sustained with 0.1 mcg/kg/min remifentanil and the dose increased to provide a MAP of 60‒65 mmHg. In Group E, esmolol was used with a loading dose of 1 mg/kg in 60 sec. The maintenance was sustained with 0.4 mg/kg/h esmolol and the dose increased to provide a MAP of 60–65 mmHg. In Group N, nitroglycerin was used with a maintenance dose of 2 mcg/min to provide a MAP of 60–65 mmHg. During the procedure HR less than 50 beats/min were defined as bradycardia and it was treated with 0.015 mg/kg atropine. In addition, if the MAP decreased to less than 60 mmHg for more than 60 s, the dosage of the drug is halved and followed further. If the intolerable hypotension continued, then the CH medication was terminated. After FESS was completed and the study drug was terminated, the patients were followed up to opening their eyes and extubation time.

During procedures; HR, SAP, DAP, MAP, SpO_2_, BIS, bleeding scores, extra drugs used were monitored in 5, 10, 15, 20, 25, 30, 40, 50, 60, 80, 100, 120 min. In addition, the same parameters were recorded in the period of time between the termination of CH and extubation. Other medications used were recorded. Bleeding Scores (BLS) were declared by the primary surgeon. (BLS-0, No bleeding; BLS-1, minimal bleeding, no need for aspiration need; BLS-2, minimal bleeding, the infrequent necessity of aspiration; BLS-3, minimal bleeding, frequent necessity of aspiration; BLS- 4, moderate bleeding, frequent necessity of aspiration; BLS-5. Severe bleeding). The total amount of the volatile agent (Sevoflurane) used was documented. The efficacy of the drugs was evaluated with LTMAP, Surgical Field Bleeding Score (SFBS) and Surgeon’s Satisfaction (SS). SS were graded as excellent, good, moderate, bad, and very bad. For analysis, SS were grouped as satisfied (good and excellent) and not satisfied (moderate, bad, very bad). To increase the efficacy and optimal subjectivity of the evaluation process, only 2 surgeons were included in the study.

Baseline characteristics of the patient group were described using proportions for dichotomous and categorical variables. Differences between continuous variables were assessed with the Student *t*-test and non-parametric tests for repeated measures (Friedman Test). Differences between non-parametric variables were analyzed with Mann–Whitney U test. The Chi-Square or Fisher exact tests were used to compare categorical variables. All analyses were performed using SPSS 17.0 for Windows (IBM Corp., Armonk, NY). The p-value of less than 0.05 was considered as statistically significant.

## Results

**Figure 1:**
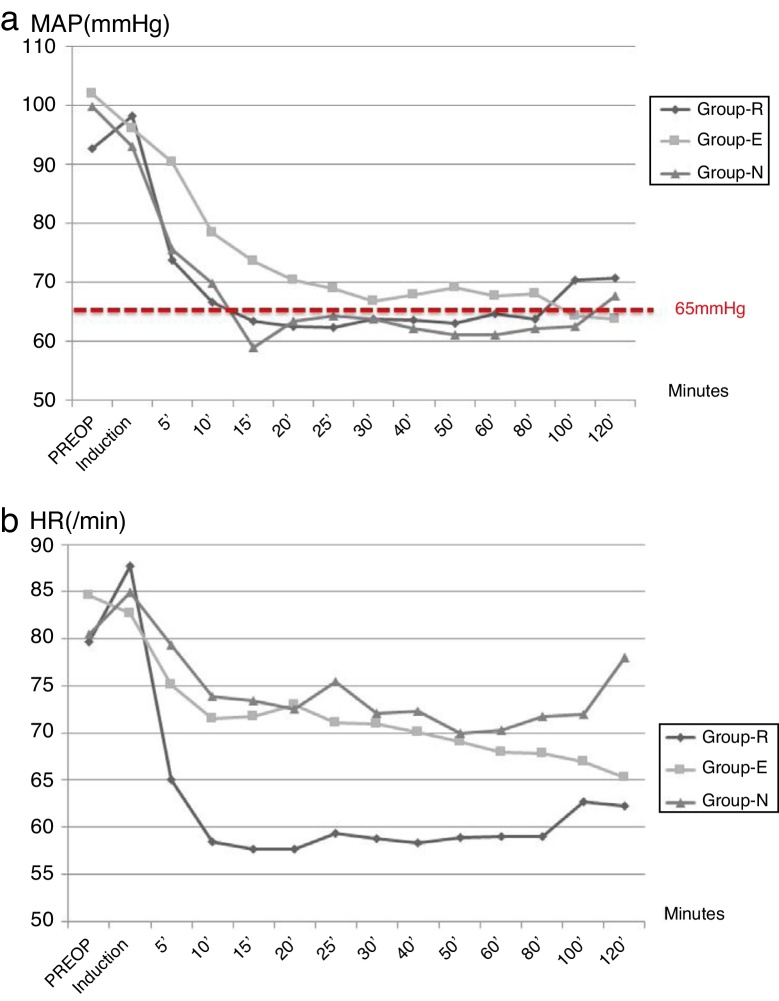
The median values of MAP and HR during CH.

Between May to December 2015, 60 patients were included and randomized into 3 different study groups. The baseline characteristics of the patients are summarized in Table 1. The median length of operations was 60 min (50–120 min.) (Table 2). The median length of operations was shorter in Group R (60 vs. 70, *p* = 0.43). In 95% of the patients, CH goals were achieved. In all study groups success rate was more than 90% and it was 100% in Group N. The median LTMAP was 10 (5–20) minutes in Group R, 15 (4–40) minutes in Group E and 15 (5–40) minutes in Group N (*p* = 0.052) (Fig. 1a). It was shorter in Group R when compared with Group E (*p* = 0.01) and Group N (*p* = 0.14). The analysis of HR concluded that the maximum HR during procedures were similar in all study arms groups (*p* = 0.90) and Group R were exposed to more bradycardia (*p* < 0.001) (Table 2) (Fig. 1b).

**Table 1:** The characteristics of patients.

Characteristics	Group R (n, range)	Group E (n, range)	Group N (n, range)	Total (n, range)	*p*
Age	36.5 (18‒56)	33.5 (20‒55)	47.5 (19‒65)	39 (18‒65)	0.02
Weight (kg)	78.0 (60‒100)	75.0 (50‒96)	74 (60‒96)	75 (50‒100)	0.90
Height (cm)	170 (161‒185)	171.5 (163‒176)	170 (160‒185)	170 (160‒178)	0.50
BMI (kg/m^2^)	26.9 (20.3‒35.0)	26.5 (18.4‒31.4)	25.4 (20.7‒32.4)	26.3 (18.4‒ 35.0)	0.83
Obese (n,%)	4 (20.0%)	4 (20.0%)	5 (25.0%)	13 (21.7%)	0.90
Male (n,%)	13(65.0)	17(85.0)	13(65.0)	43(%71.7)	0.26
ASA score					0.62
ASA 1	10 (50.0%)	8 (40.0%)	11 (55.0%)	29 (48.3%)	
ASA 2	10(50.0%)	12(60.0%)	9(45.0%)	31(51.7%)	
Preoperative SBP (mmHg)	135 (118‒157)	135 (110‒152)	133 (117‒155)	135 (110‒157)	0.92
Preoperative DBP (mmHg)	76 (61‒89)	85 (60‒102)	77 (60‒107)	78 (60‒107)	0.02
Preoperative MAP (mmHg)	96.5 (83‒111)	103.5 (82‒117)	100 (88‒120)	100 (82‒120)	0.41
Preoperative HR (beats/hour)	79.7 (51‒107)	87.5 (61‒107)	84 (55‒100)	81 (51‒ 107)	0.61
Preoperative SpO2 (%)	96 (92‒98)	97.5 (93‒100)	97 (91‒100)	97 (92‒100)	0.19

ASA, American society of anesthesiologists; DBP, diastolic blood pressure; HR, heart rate; MAP, Mean arterial pressure; SBP, systolic blood pressure; SpO2, Capillary oxygen saturation; BMI, Body mass index.

**Table 2:** The parameters under FESS and CH in study arms.

	Group R (median, range)	Group E (median, range)	Group N (median, range)	*p*
Length of FESS (min)	60 (50‒120)	70 (60‒120)	70 (60‒120)	0.77
Success of CH	19 (95.0%)	18 (90.0%)	20 (100%)	0.34
LTMAP (min)	10 (5‒20)	15 (5‒40)	15 (5‒40)	0.052
MAP difference between baseline and target (mmHg)	36 (16‒49)	39 (18‒52)	39.5 (23‒56)	0.61
Maximum HR (beats/min)	94.5 (65‒107)	94.0 (77‒107)	92.5 (76‒105)	0.90
Minimum HR (beats/min)	52.5 (36‒59)	63.5 (45‒74)	64.0 (54‒80)	<0.001

During the perioperative period, extra medications were used in 10 (50%) patients in Group N, 7 (35%) in Group R and 3 (15%) patients in Group E (*p* = 0.051) for hemodynamic changes (Table 3). In addition, medications used after termination of CH were also similar between groups. The amounts of sevoflurane as a volatile anesthetic used were 25.0 mL (15–51), 43.0 mL (21–105) and 40.0 mL (26–97) in Groups R, E and N, respectively (*p* < 0.001). There were postoperative complications in 10% of Group R, 5% of Group E and none of the Group N patients (*p* = 0.34). Bronchospasm was observed as a complication in these groups.

**Table 3:** The parameters evaluating the efficacy of groups.

	Group R (n,%)	Group E (n,%)	Group N (n,%)	*p*
Volatile use (Sevoflurane), mL (median, range)	25.0 (15‒51)	43.0 (21‒105)	40.0 (26‒97)	<0.001
Extra medication for hemodynamic changes	7 (35.0)	3 (15.0)	10 (50)	0.062
Extra medication after termination of CH	1 (5.0)	0 (0)	0 (0)	0.36
Postoperative complications	2 (10)	1 (5)	0 (0)	0.34
Maximum SFBS (median, range)	2 (1‒2)	2 (2‒3)	3 (2‒4)	<0.001
Surgeon’s satisfaction				<0.001
Very bad	0 (0)	0 (0)	1 (5)	
Bad	0 (0)	0 (0)	5 (25)	
Moderate	0 (0)	8 (40)	8 (40)	
Good	10(50)	9 (45)	6 (30)	
Excellent	10 (50)	3(15)	0 (0)	
Maximum MAP after termination of CH mmHg (median, range)	100.5 (67‒137)	85.0 (65‒113)	94.0 (68‒116)	0.21

The analysis of efficacy was performed by comparing LTMAP, SFBS, and SS in study arms. The SFBS were 2 (1–2) in Group R, 2 (2–3) in Group E and 3 (2–4) in Group N (*p* < 0.001). While there was more bleeding with nitroglycerin, SFBS scores were less in Group R when compared with esmolol (*p* = 0.001) and nitroglycerin (*p* < 0.001). The analysis of SS scores concluded that surgeons were more satisfied with Group R (100%), when compared with Group E (60%) and Group N (30%) (*p* < 0.001). There was a positive correlation with SFBS and SS scores (r = 0.43, *p* < 0.001).

## Discussion

In the present study, we aimed to compare the efficacy of three CH agents, Remifentanil, esmolol, nitroglycerin. We concluded that less volatile agent, short time to approach CH, stable blood pressure, less SFBS and short operation time length were the advantages of Remifentanil. Efficacy of approaching targeted MAP and less postoperative complications were the advantages of Nitroglycerin.

Functional endoscopic sinus surgery is widely used because of its feasibility and low complication rates. Bleeding is the most common complication of FESS.^6^ In addition to other surgical procedures, CH is also integrated with FESS to decrease the bleeding during FESS and provide better surgical field.^7^ Numerous agents are used for CH. The ideal hypotensive agent should be easy to administer and safe. In addition, it should have a rapid onset of action, short half-life, easily predictable and observable side effects.^8^, ^9^ The most commonly used agents are magnesium sulfate, vasodilators (sodium nitroprusside), nitroglycerin, potent inhaled anesthetics and Beta-adrenergic antagonists.^5^ There are both pros and cons for these agents and there are numerous data about comparing these agents. In the present study we tried to compare three most commonly used medications in CH and evaluate their efficacy in FESS: remifentanil (μ-opioid receptor agonist), esmolol (short-acting β-adrenergic receptor blocker) and nitroglycerin (vasodilator).

In the literature, there are numerous studies comparing 2 drugs, especially esmolol and nitroglycerin. However, there are limited studies comparing three agents for CH. Srivastava et al. concluded that nitroglycerin was superior to esmolol with its shorter LTMAP, lower bleeding scores and producing less reflex tachycardia.^10^ In addition, the study which compared esmolol and nitroglycerin in nasal surgery showed that esmolol provided more hemodynamic stability and better surgical field control.^11^ Our results were consistent with the data in the literature. There was more hypotension, worse SFBS, more need for extra medication and worse SS scores in the nitroglycerin group.

Esmolol is a β-adrenergic receptor blocker and has been used for CH for many years. In addition, vasoconstriction in arterioles and precapillary sphincters provides less bleeding and better operation field.^12^ The efficacy of esmolol in CH has been compared with others in numerous studies. Degoute et al. compared esmolol, Remifentanil, and nitroprusside and concluded that esmolol was more effective for decreasing middle ear blood flow.^13^ Pilli et al. also showed the efficacy and safety of esmolol in CH.^14^ In our study, esmolol provided effective CH and stable hemodynamic parameters during the FESS.

Esmolol and nitroglycerin provide hypotension by directly acting on cardiovascular structures. However, Remifentanil is an ultra-short-acting µ-agonist opioid receptor. Its most important advantages are short half-life and not having effects on microcirculation.^5^, ^6^, ^7^, ^8^, ^9^, ^10^, ^11^, ^12^, ^13^, ^14^, ^15^ As a CH agent, its superiority over fentanyl and sufentanil has been reported.^1^ Although the efficacy of Remifentanil has been documented in our study, it has a dose-dependent, depression effect on the sinoatrial node. The studies, comparing Remifentanil with others, documented more bradycardia with Remifentanil.^16^, ^17^ Consequently, it is recommended to be avoided in patients with a cardiac dysfunction or risk of bradyarrhythmia.^18^, ^19^ In our study, we observed lower HRs in Group R and in 3 (15%) patients were treated with atropine.

The study had some inevitable limitations. The evaluation of CH efficacy was performed by assessing SS and SFBS. However, those are subjective parameters. We tried to decrease the bias related with subjectivity by working with 2 surgeons. Objective parameters could provide a more efficient analysis. The inclusion of more patients could enable us to do further analysis. The patients were older in the Group N when compared with others. However, because the renal, hepatic functions and performance scores of the patients were similar, we ignored the age difference between groups.

## Conclusion

Remifentanil provides a lower surgical field bleeding score, stable blood pressure and short time to targeted mean arterial blood pressure with the use of less volatile anesthetic agent. However the nitroglycerin group produces easy control of blood pressure with less postoperative complications. As a result, appropriate CH agents should be selected according to patients’ characteristics and advantages/disadvantages of drugs during FESS procedures.

## Conflicts of interest

The authors declare no conflicts of interest.
